# Editor's Note on ‘Cancer-derived p53 mutants suppress p53-target gene expression—potential mechanism for gain of function of mutant p53’

**DOI:** 10.1093/nar/gkac1265

**Published:** 2023-01-10

The Editors were first alerted in late 2013 about potential issues with Figures 1A and 3I of *Nucleic Acids Research (NAR)*, Volume 35, Issue 6, 15 March 2007, Pages 2093–2104, https://doi.org/10.1093/nar/gkm099. The journal investigated the matter at the time and did not find conclusive evidence to support the allegations.

When similar and new allegations about Figures 1C-D and Figure 2A were brought to the Editors’ attention again in late 2021, the journal re-opened the investigation. A summary of the allegations and conclusions based on the authors' response is provided below.

- Figure 1A: The pig3 bands are similar.
- Figure 1C: The T47D cells/pig3 and CNE-2 cells/pig3 bands are similar after vertical flip.

The authors no longer have the original data but have provided a composite RT-PCR figure for the data used in Figures 1A-C. This is another set of PCRs that were conducted with the same samples, confirming the results.


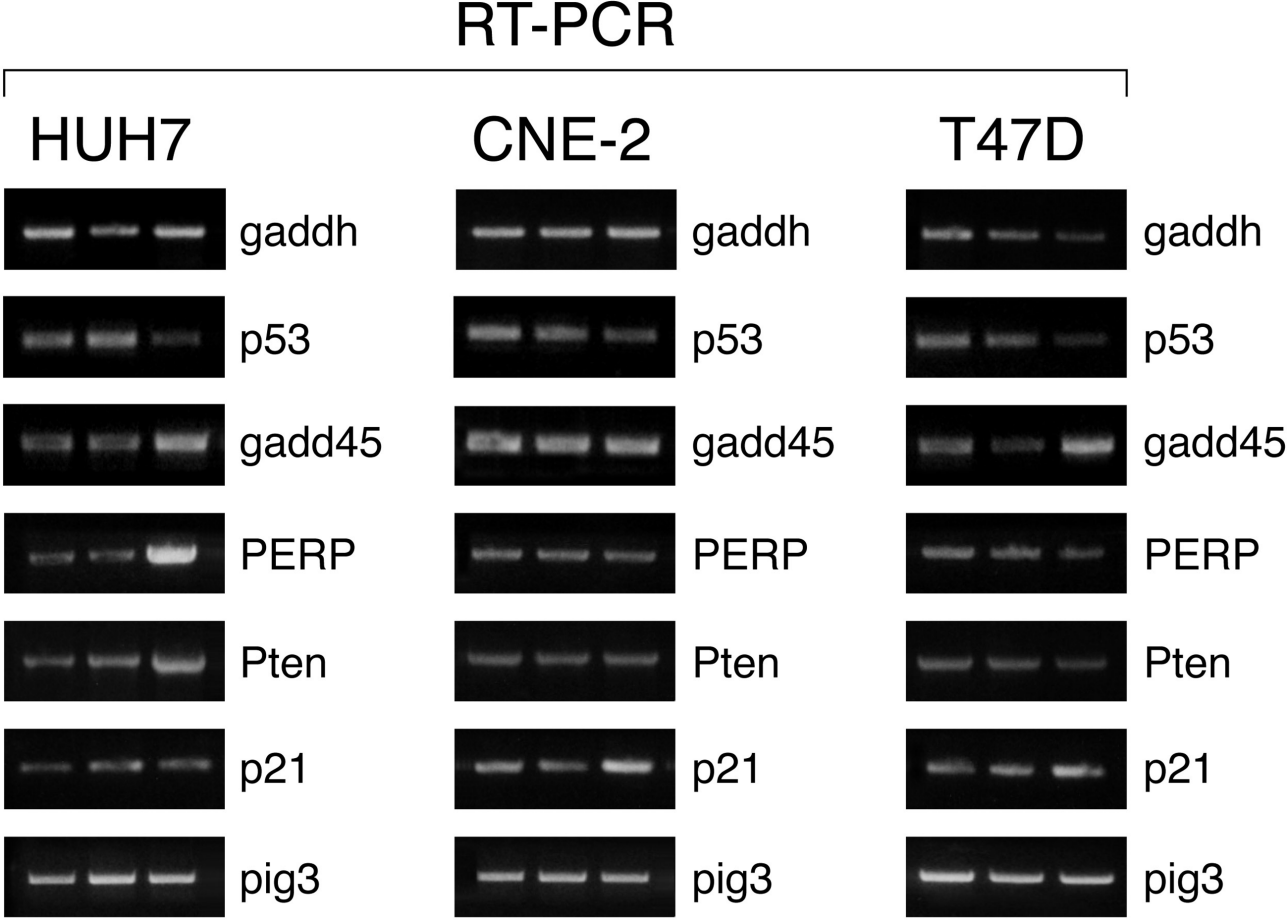

- Figure 1D: sign of splicing in the Tap73 panel.

The authors no longer have the original data but acknowledge that the samples in the experiment TAp73 may have been in a different order and the bands assembled to match the other experiments.

- Figure 2A, lower gapd panel: bands 7 (245 Arg) and 13 (273 Arg) are similar, bands 10 (249 Pro) and 12 (273 Pro) are also similar.

The authors have provided the original image, as shown below, confirming the results.


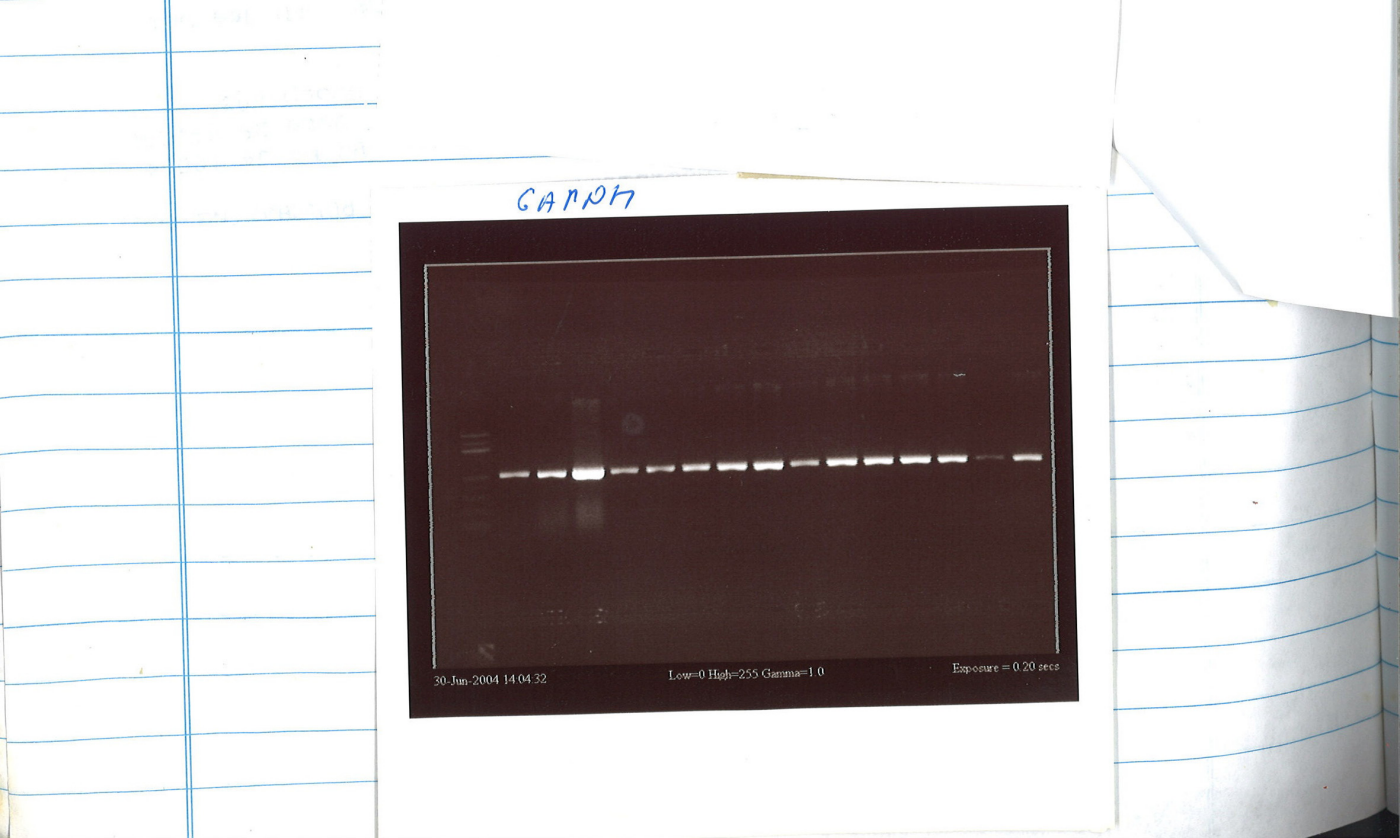

- Figure 3I: This panel is identical to Figure 2 of Vikhanskaya et al. 2005, with several of the same co-authors as the *NAR* article, herein referred to as (1). However:
  - Figure 3I does not acknowledge Figure 2 of (1) as the original publication.
  - p53: The last 2 bands in the *NAR* panel (WT) are not included in (1) and were most likely added at a later stage.
  - Actin: The first 12 lanes are identical, while lane 13 is different (282 Arg). The last 2 lanes, 14–15, are not present in Figure 2 of (1).

The authors no longer have the original data.


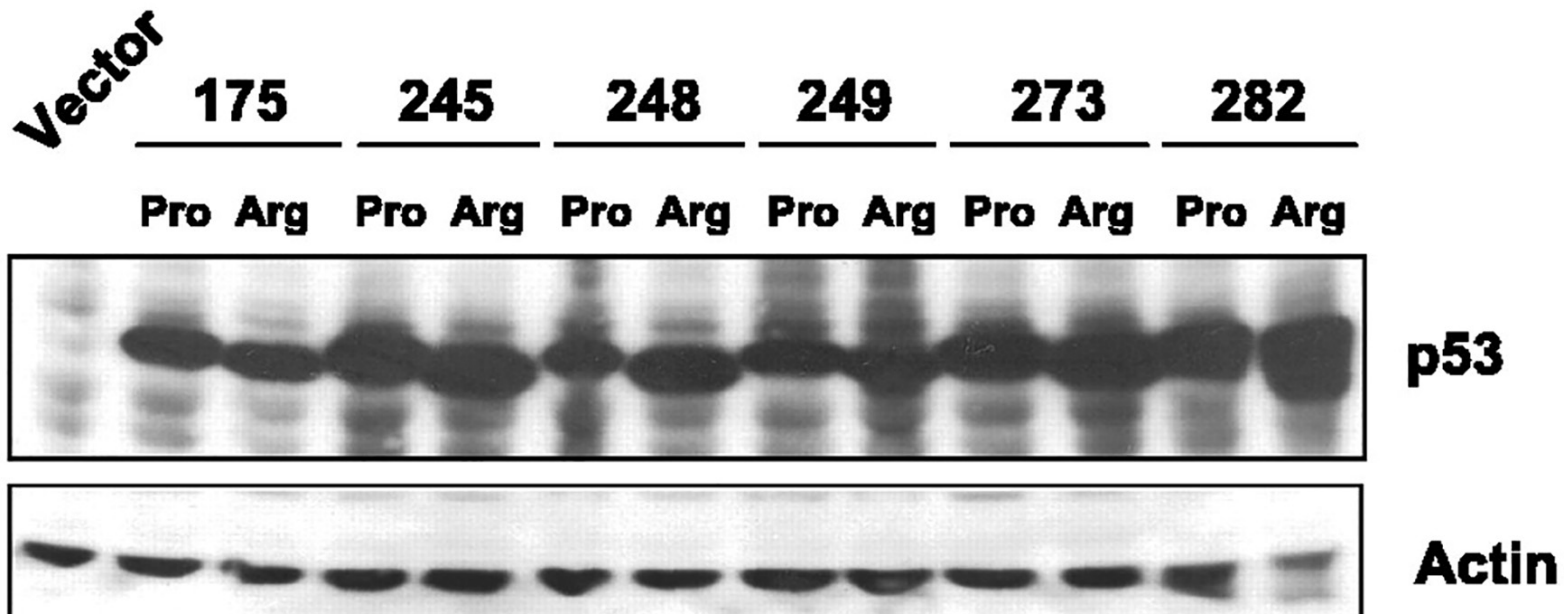



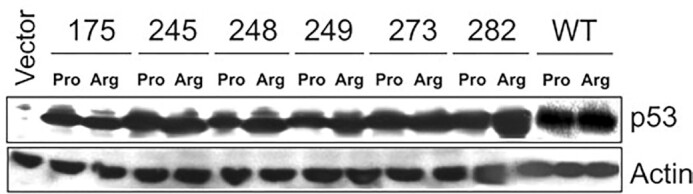


**Original Figures**. Upper panel is Figure 2 from (1), lower panel is Figure 3I from *NAR*.


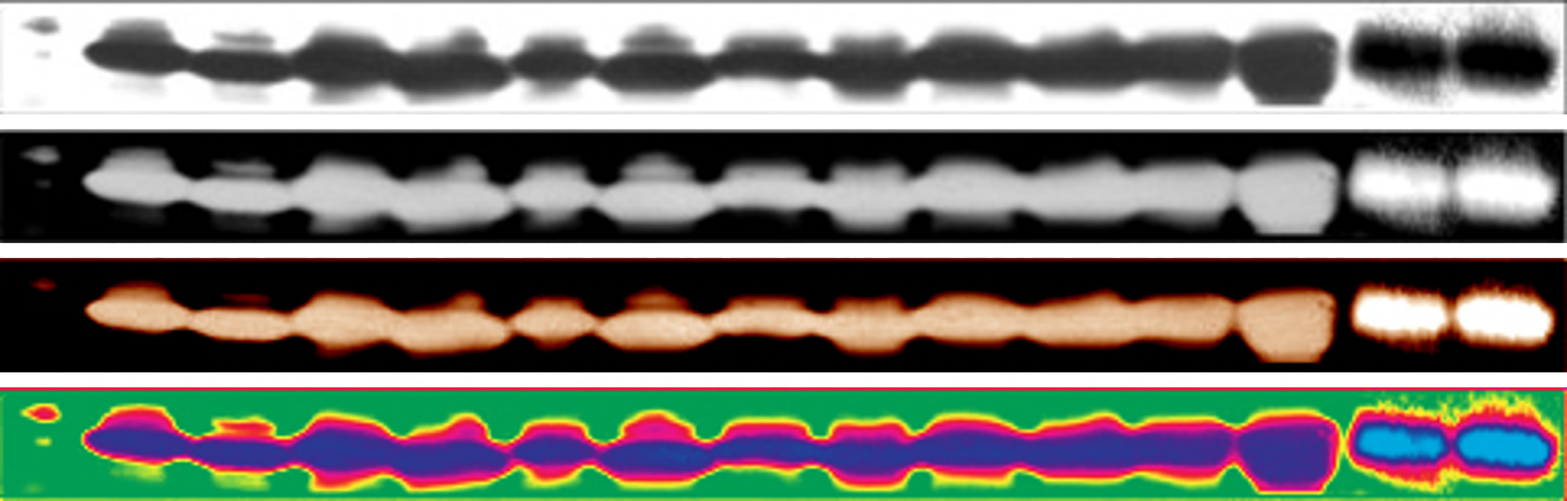


***NAR* p53 panel**. From top to bottom: original panel, negative, colour negative, gradient map. The last 2 lanes are different and most likely come from a different experiment.


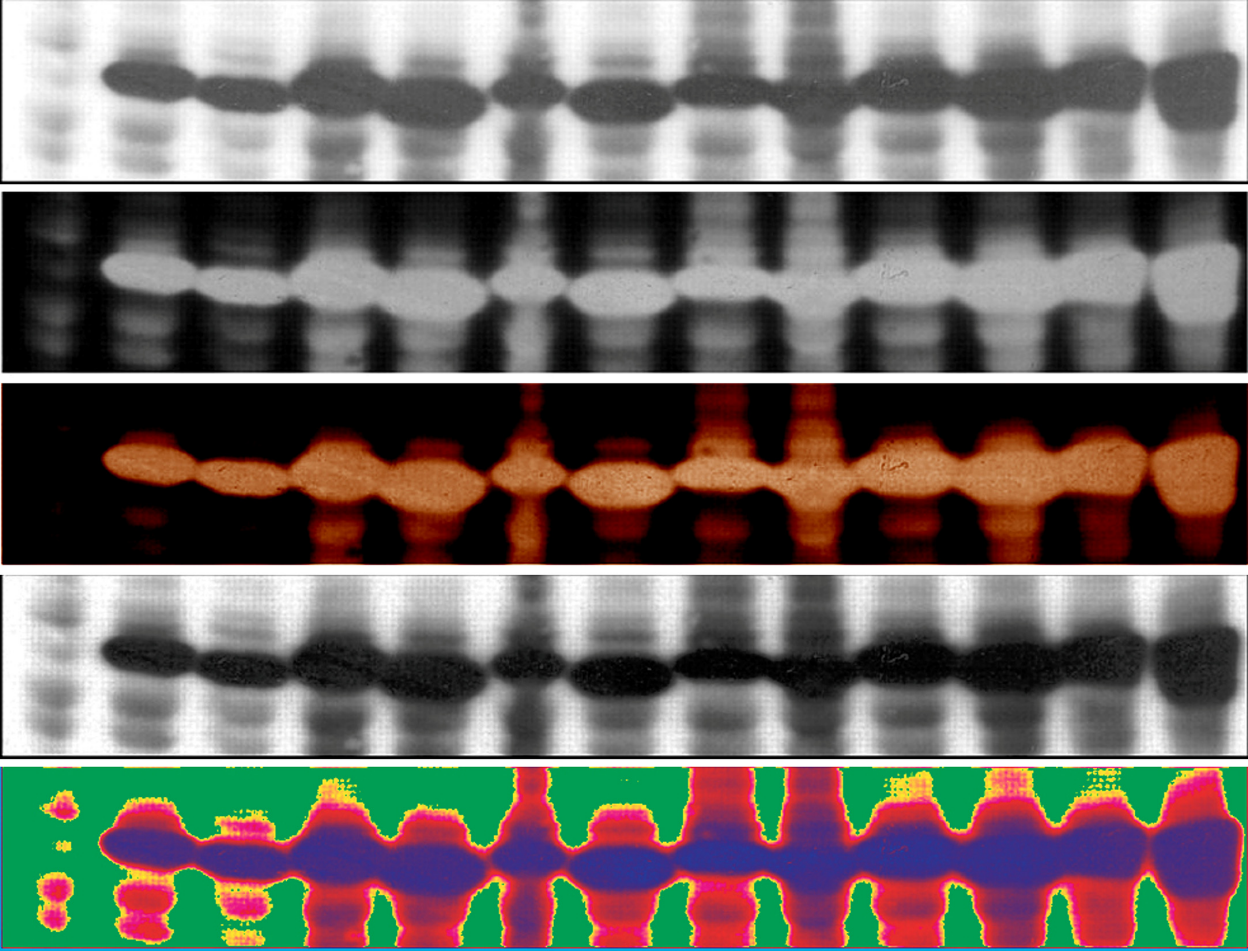


**p53 panel from (1)**. From top to bottom: original panel, negative, colour negative, equalise adjustment, gradient map.


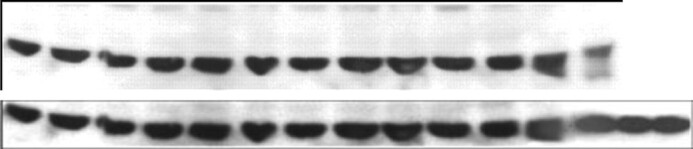


**Actin panels**. Upper panel from (1) after size, brightness and contrast adjustments. Lower panel from *NAR* Figure 3I. The first 12 bands are identical, band 13 differs and bands 14–15 are not present in the upper panel. Bands 13–15 most likely come from a different experiment.


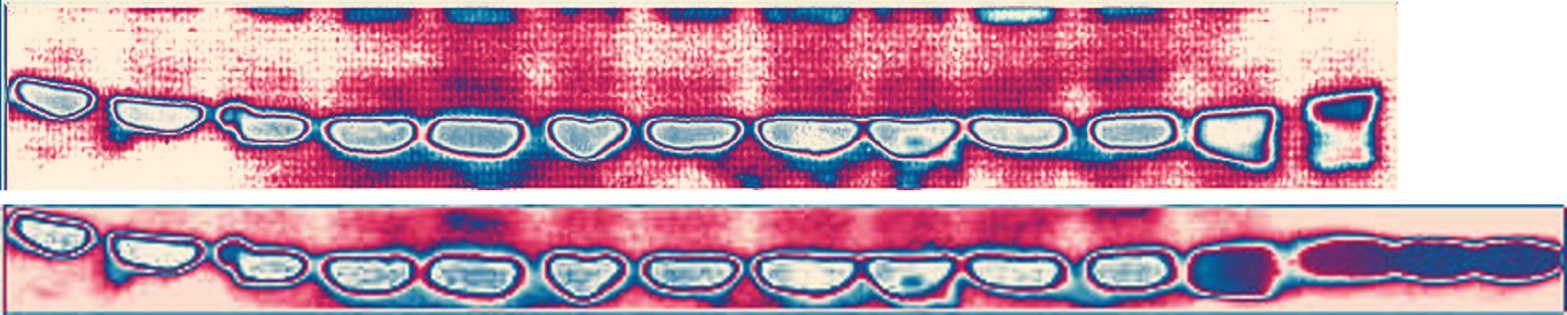


**Gradient maps of the actin panels**. Upper panel from (1), lower panel from *NAR* Figure 3I. The first 12 bands are identical, band 13 differs, and bands 14–15 are not present in the upper panel. Bands 13–15 most likely come from a different experiment.

In conclusion Figure 3I has been recycled from (1) without proper attribution. In the actin panel, the first 12 samples are identical. The sample in lane 13, 282 Arg, is different and may have no relation to the corresponding p53 panel. While these issues may not affect the results or conclusion of the study, in the absence of original data, the Editors advise readers to examine the figure with care.

Julian E. Sale, Barry L. Stoddard

Senior Executive Editors
